# Horizontal versus Familial Transmission of *Helicobacter pylori*

**DOI:** 10.1371/journal.ppat.1000180

**Published:** 2008-10-24

**Authors:** Sandra Schwarz, Giovanna Morelli, Barica Kusecek, Andrea Manica, Francois Balloux, Robert J. Owen, David Y. Graham, Schalk van der Merwe, Mark Achtman, Sebastian Suerbaum

**Affiliations:** 1 Institute of Medical Microbiology and Hospital Epidemiology, Hanover Medical School, Hanover, Germany; 2 Department of Molecular Biology, Max Planck Institute for Infection Biology, Berlin, Germany; 3 Department of Zoology, University of Cambridge, Cambridge, United Kingdom; 4 MRC Centre for Outbreak Analysis and Modelling, Department of Infectious Disease Epidemiology, Imperial College Faculty of Medicine, St Mary's Campus, London, United Kingdom; 5 Campylobacter and Helicobacter Research/Reference Unit, Laboratory of Enteric Pathogens, Centre for Infections, Health Protection Agency, London, United Kingdom; 6 Department of Medicine, Veterans Affairs Medical Center, Baylor College of Medicine, Houston, Texas, United States of America; 7 Hepatology and Gastroenterology Research Unit, Department of Internal Medicine, University of Pretoria, Pretoria, South Africa; 8 Environmental Research Institute and Department of Microbiology, University College Cork, Cork, Ireland; University of Oxford, United Kingdom

## Abstract

Transmission of *Helicobacter pylori* is thought to occur mainly during childhood, and predominantly within families. However, due to the difficulty of obtaining *H. pylori* isolates from large population samples and to the extensive genetic diversity between isolates, the transmission and spread of *H. pylori* remain poorly understood. We studied the genetic relationships of *H. pylori* isolated from 52 individuals of two large families living in a rural community in South Africa and from 43 individuals of 11 families living in urban settings in the United Kingdom, the United States, Korea, and Colombia. A 3,406 bp multilocus sequence haplotype was determined for a total of 142 *H. pylori* isolates. Isolates were assigned to biogeographic populations, and recent transmission was measured as the occurrence of non-unique isolates, i.e., isolates whose sequences were identical to those of other isolates. Members of urban families were almost always infected with isolates from the biogeographic population that is common in their location. Non-unique isolates were frequent in urban families, consistent with familial transmission between parents and children or between siblings. In contrast, the diversity of *H. pylori* in the South African families was much more extensive, and four distinct biogeographic populations circulated in this area. Non-unique isolates were less frequent in South African families, and there was no significant correlation between kinship and similarity of *H. pylori* sequences. However, individuals who lived in the same household did have an increased probability of carrying the same non-unique isolates of *H. pylori*, independent of kinship. We conclude that patterns of spread of *H. pylori* under conditions of high prevalence, such as the rural South African families, differ from those in developed countries. Horizontal transmission occurs frequently between persons who do not belong to a core family, blurring the pattern of familial transmission that is typical of developed countries. Predominantly familial transmission in urban societies is likely a result of modern living conditions with good sanitation and where physical contact between persons outside the core family is limited and regulated by societal rules. The patterns observed in rural South African families may be representative of large parts of the developing world.

## Author Summary

More than half of the world population is infected with *Helicobacter pylori*. It was widely believed that the primary mode of transmission is intra-familial, but the chains of infection are poorly understood. We have applied multilocus sequence analysis to *H. pylori* from two large multi-generation families in rural South Africa. Observations were compared with *H. pylori* from small, nuclear families in urban areas of the United States, United Kingdom, Colombia, and Korea, as well as with a large global collection from unrelated individuals. Intra-familial transmission of *H. pylori* was common in urban families but less common in the rural South African families. Instead, the South African families were infected with widely diverse strains, and multiple infections were common. We argue that the apparent predominance of intra-familial transmission in urban societies is a result of good socioeconomic conditions. In high-prevalence areas, opportunities for horizontal transmission are higher, which can result in greater diversity of *H. pylori* within a family. The patterns of frequent horizontal spread in rural South African families may be representative of large parts of the developing world. This interpretation is supported by the global sample which yielded ample evidence for horizontal inter-familial transmission in many areas of the world.

## Introduction

*Helicobacter pylori* infects an estimated 50% of the human population, and can result in chronic gastritis, gastric or duodenal ulcers, gastric cancer, and MALT lymphoma [1]. Genetic diversity in *H. pylori* is high among isolates of similar geographic origin and is even higher on a global scale [2]. Biogeographic differences between these organism are thought to reflect both ancient and more recent human migrations [3], unlike most other human pathogens where horizontal transmission is so efficient, even at global scales, that geographic associations are rare or only transient [4]. The biogeographical associations within *H. pylori* are thought to reflect transmission within families, or local communities, and are thought to largely depend on person to person contact.

The term “vertical transmission” has been used to describe *H. pylori* transmission patterns [5],[6], but to avoid confusion with traditional uses of this term that are restricted to the transmission from mother to child in the perinatal period, we prefer to use the term “familial” to summarize transmission between parents and children as well as transmission between siblings. After more than 20 years of research, remarkably little is known about the details of the modes of transmission of *H. pylori* and its routes of spread. The primary modes of transmission are thought to be fecal-oral and oral-oral (e.g. via vomitus) but some indirect evidence has also been published for transmission via drinking water and other environmental sources (for recent reviews, see [7],[8]).

Primary reasons for the paucity of information about the mode of transmission of *H. pylori* include the difficulty of sampling *H. pylori*, which requires the sampling of gastric contents and the culture of a fastidious bacterium, at a community level and the very high genetic diversity and variability of *H. pylori* that cannot be satisfactorily addressed with classical high-throughput forms of analyses that depend on molecular fingerprinting (for a review see [9]). The unusual combination of a high mutation rate plus a high frequency of homologous recombination has generated so much diversity that most multilocus sequences are unique in samples of *H. pylori* from unrelated humans [3], [10]–[12]. Population genetic tools based on such multilocus sequences have allowed the assignment of individual isolates to one of six discrete biogeographic bacterial populations but this level of resolution is inadequate to address questions of local transmission. Instead, most isolates from a continental area such as Europe or East Asia belong to a single biogeographic population, which have been designated hpEurope and hpEastAsia, respectively. In other geographic areas, isolates have been assigned to the bacterial populations hpAsia2, hpNEAfrica, hpAfrica1 and hpAfrica2, whose designations reflect their geographic sources. Where multiple populations have been identified in a single locale, such as the Americas, these are thought to have arisen through secondary human migrations in recent centuries or millennia [3],[13]. Consistent with such an explanation, isolates of *H. pylori* in Cape Town, South Africa belonged to hpAfrica1, which was thought to have been introduced by the Bantu migrations, plus hpEurope, which are thought to have been introduced by European colonists [3]. However, a few other isolates were assigned to the highly distinct hpAfrica2, whose sources and history remain unclear.

The concept that transmission of *H. pylori* is predominantly familial is based on fingerprinting and sequencing studies that documented the clonal spread of *H. pylori* infection within several families [11], [14]–[19]. Until now, most such studies were performed with nuclear families consisting of parents and their children from industrialized, urban sources. The frequency of infection by *H. pylori* in industrialized settings is decreasing, suggesting that transmission may be relatively rare in such environments. We therefore used multilocus sequence analysis to contrast the patterns of familial transmission from a variety of urban sources with the transmission patterns in two large multi-generation families from rural South Africa. The data from the urban families of non-South African origin confirmed previous findings of frequent clonal transmission of *H. pylori* between first degree relatives. Very different results were obtained in rural South Africa, where horizontal transmission between unrelated individuals seems to play an equally important role.

## Results

### *H. pylori* from rural families in South Africa

**Figure 1:**
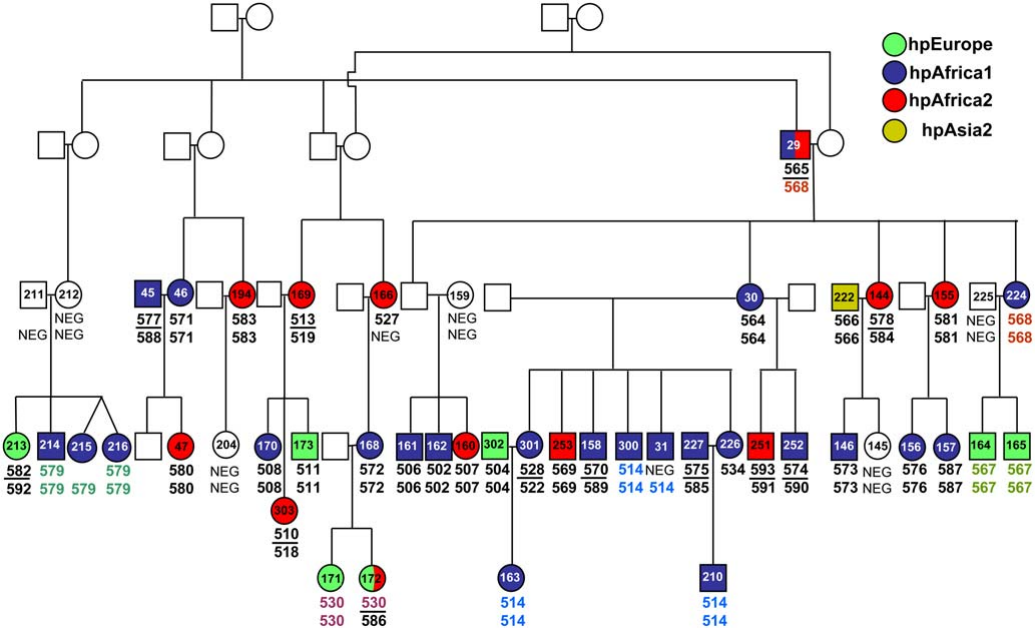
*Helicobacter pylori* in family 12 from South Africa. Circles in the pedigree depict female and squares depict male family members. Numbers in the symbols refer to the patient/isolate identifyer. Empty symbols: no biopsies were taken, because individuals refused to participate or were deceased. Colours of filled symbols indicate the assignment of the isolate to *H. pylori* populations by Structure 2.0 analysis. Color-coding is shown on the right. Numbers below the symbols indicate the sequence type (ST) of the antrum (upper number) and corpus (lower number) isolates. STs that occurred in more than one individual are highlighted by the same colour. Black horizontal lines between upper and lower ST numbers indicate that antrum and corpus isolates were assigned to different STs. NEG: *H. pylori* could not be cultivated from the respective biopsy. Individuals where the upper or lower number are missing indicate that only one biopsy was available.

**Figure 2:**
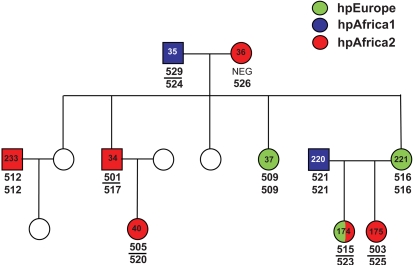
*Helicobacter pylori* in family 13 from South Africa. See legend of Figure 1 for details.

We determined the genetic relationships between *H. pylori* that infected two multi-generation families living in a rural area near Pretoria, South Africa. Gastric biopsies were taken from both the antrum and corpus of 45 members of family 12 (Figure 1) and 10 members of family 13 (Figure 2). Additional single biopsies from either antrum or corpus were obtained from three other members of family 12. *H. pylori* was cultivated from 90% of these individuals, resulting in a total of 99 isolates from 52 individuals: paired isolates from antrum and corpus of 47 individuals, and a single isolate from either corpus or antrum from five individuals. Each isolate was subjected to multilocus sequence typing, resulting in a multilocus haplotype of 3,406 bp after concatenation of the sequences of fragments of seven housekeeping genes. These haplotypes were then assigned to one of the four modern *H. pylori* populations hpEurope, hpAfrica1, hpAfrica2 or hpAsia2 [3],[12] by the Bayesian program Structure 2.0 (Figures 1 and 2). None of the haplotypes from these families belonged to the hpEastAsia or hpNEAfrica populations.

These results resemble those previously described from Cape Town, where *H. pylori* belonged to hpAfrica1, hpAfrica2 and hpEurope [3]. It was nevertheless unexpected that 18/99 isolates were hpEurope in a rural African village because hpEurope bacteria are thought to have originally been imported from Europe but none of the inhabitants of the village was of European origin. Two of the 99 isolates were hpAsia2, and presumably were originally imported from Asia. Half (48/99) of the isolates were hpAfrica1, which is thought to have accompanied the Bantu migrations from West Africa. Finally, this analysis identified an additional 31 hpAfrica2 isolates in the two multi-generation families whereas previously only ten hpAfrica2 isolates had been described, all from individuals with mixed ancestry and whites in Cape Town. Thus, this rural community contained a mixture of *H. pylori* from both African (hpAfrica1, hpAfrica2) and non-African (hpEurope, hpAsia2) sources. And this mixture of origins was also accompanied by multiple infections of individuals, because *H. pylori* of distinct populations were isolated from the antrum and corpus of three individuals (hpAfrica1/hpAfrica2 in one case and hpAfrica2/hpEurope in two others).

In order to trace patterns of transmission within the families, we also analyzed the same data by the multilocus sequence typing approach [20], according to which each unique allele receives a distinctive numeric designation and each unique combination of alleles at the seven loci is assigned a distinctive numeric sequence type designation. The 99 isolates from the 52 members of families 12 and 13 fell into a total of 56 unique STs and five non-unique STs. We use the designation non-unique ST for any ST that was isolated from more than one individual and consider the existence of non-unique STs as presumptive evidence for transmission between the individuals from whom it was isolated. The five non-unique STs were all isolated from family 12. Three were isolated from siblings (SA214/SA215/SA216, SA171/SA172, SA164/SA165). A fourth non-unique ST was isolated from a pair of siblings (SA300/SA31) plus their niece (SA163) and nephew (SA210). The fifth non-unique ST was from a father (SA29) and his child (SA224) (Figure 1). These results suggested that transmission did occur between first degree relatives in the South African families but that such transmission was not as common as might have been expected if familial transmission were the rule for *H. pylori*. We therefore examined two additional sources of data, namely global samples of *H. pylori* that are not known to have been isolated from family members and isolates of *H. pylori* from urban families that are similar to those that have been analyzed in the past.

### Frequencies of non-unique isolates within a global sample

We have interpreted the frequency of non-unique STs among first degree relatives in the South African families as an indication of familial transmission. If that interpretation is correct, then non-unique isolates should be less frequent in population samples which are not known to include first degree relatives. Data for such population samples have been published for 769 isolates from 51 locations [12] but many of these locations were urban and might not be comparable to the rural South African families analyzed here. We have therefore examined the data for all 1,852 isolates in our databases which were obtained from a total of 97 locations (Table S1). Many of the additional 1,087 isolates were from native populations, and many of the additional 48 locations were rural settings in Namibia, Siberia, East Asia, the Pacific Islands and the former continent called Sahul, consisting of modern Australia plus Papua New Guinea (manuscripts in preparation). The frequencies of non-unique isolates were significantly lower in the global sample than among isolates from first-degree relatives in the South African families (Tables 1 and S2). However, when uniqueness was defined by less stringent criteria (identity at only five or six of the seven alleles or based on DNA homologies of the concatenated sequences of 99–99.95%), the frequencies of non-unique isolates in the South African families were not significantly different from the global sample. These data suggest that horizontal transmission is a relatively frequent phenomenon among individuals that are not first degree relatives in some rural areas. Such transmission may be between distantly related or unrelated individuals, or from a common environmental source. The highest frequencies under relaxed criteria of non-unique isolates were within samples from native inhabitants in the Americas and in the Sahul, whereas non-unique isolates were rare within samples from the Middle East and Europe, even when relaxed criteria were used.

**Table 1:** Non-unique *H. pylori* isolates based on numbers of shared alleles in first-degree relatives from South Africa and urban sources versus a global sample of isolates.

Sources	Numbers of non-unique isolates		
(number of isolates)	7/7	6/7	5/7
1. rural South African families (68)	11 (0.16)	13 (0.19)	13 (0.19)
2. urban families (43)	15 (0.35)	22 (0.51)	28 (0.65)
3. global, not known to be related (1852)	151 (0.08)	262 (0.14)	339 (0.18)
*X* ^2^ 1 vs. 2	4.15	11.09	21.99
*X* ^2^ 1 vs. 3	4.39	0.95	0.02
*X* ^2^ 2 vs. 3	34.3	42.3	56.0
*P* 1 vs. 2	0.04	0.0009	2.7×10^−6^
*P* 1 vs. 3	0.04	0.33	0.99
*P* 2 vs. 3	5×10^−9^	8×10^−11^	7×10^−14^

### Limited diversity and frequent transmission within urban families of non-South African origin

Although significantly higher than the global sample, the frequencies of non-unique isolates in the South African families were lower than expected from previous evidence for familial transmission [11], where transmission between parents and children seemed to be the predominant mode [14]–[19]. These prior studies used fingerprinting, or sequencing of a limited number of genes, and it was possible that apparently rare familial transmission within South African families simply reflected an increased resolution associated with sequencing of seven gene fragments. Therefore, we sequenced the same seven gene fragments for 43 isolates from 11 urban families in the United States (3 families), Northern Ireland (1), England (1), Korea (3), and Colombia (3) where preliminary evidence for intra-familial transmission had been previously reported [15],[21].

**Figure 3:**
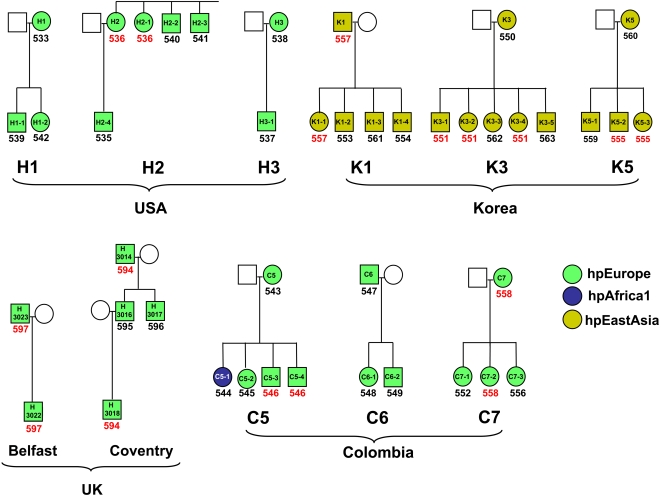
*Helicobacter pylori* in 11 families from the United States, Korea, the United Kingdom, and Colombia. Meaning of symbols and color-coding as described for Figures 1 and 2. Only one isolate was available per individual. Unique STs are shown in black and identical STs detected within a family are highlighted in red.

As expected from their geographic locations, and in contrast to the South African families, almost all *H. pylori* that were isolated from each of the urban families belonged to a single population, namely hpEurope for the families in the U.S.A., England, Northern Ireland and Colombia and hpEastAsia for the families in Korea (Figure 3). (An exceptional hpAfrica1 isolate was found in the C5 family from Colombia.) Furthermore, unlike the South African families, and in concordance with prior conclusions [11], [14]–[19], at least two first degree relatives carried isolates of the same ST within 8/11 urban families. In four of these families, the patterns apparently reflected trans-generational transmission because the same STs were isolated from a parent plus a child (families K1, Ireland, and C7), or from a grandfather and his grandson (family England, 2^nd^ degree relatives). In four other families, similar to the results from the South African families, identical STs were isolated from siblings (families H2, K3, K5, and C5) but even these may have reflected trans-generational transmission because *H. pylori* was only available from one parent per family and only one isolate was tested per individual. Three families (H1, H3 and C6) did not yield any non-unique STs (Figure 3).

The frequencies of non-unique STs among first degree relatives were significantly greater than the frequencies from the South African families or the global sample, even when relaxed criteria of identity were used (Tables 1 and 2). Thus, these results indicate that transmission within families was indeed very frequent within the urban families and often reflects transmission between parent and child. However, at least 35% of the STs were unique, even using relaxed criteria of identity, indicating that horizontal transmission from outside the family is also a major source of infection in urban settings.

**Table 2:** Non-unique *H. pylori* isolates based on percentage homology in first-degree relatives from South Africa and urban sources versus a global sample of isolates.

Sources	Numbers of non-unique isolates			
(number of isolates)	99.95%	99.9%	99.5%	99%
1. rural South African families (68)	13 (0.19)	13 (0.19)	13 (0.19)	15 (0.22)
2. urban families (43)	23 (0.53)	23 (0.53)	26 (0.60)	26 (0.60)
3. global, not known to be related (1852)	214 (0.12)	251 (0.14)	350 (0.19)	436 (0.24)
*X* ^2^ 1 vs. 2	12.7	12.7	18.0	15.1
*X* ^2^ 1 vs. 3	2.9	1.3	0.01	0.02
*X* ^2^ 2 vs. 3	63.8	51.0	43.1	29.1
*P* 1 vs. 2	0.0004	0.0004	2×10^−5^	0.0001
*P* 1 vs. 3	0.09	0.26	0.91	0.89
*P* 2 vs. 3	1×10^−15^	9×10^−13^	5×10^−11^	7×10^−8^

### Transmission patterns of *H. pylori* in South African family 12

The large number of *H. pylori* isolates available from members of family 12 permitted a test of additional hypotheses regarding intra-familial transmission pathways. We first asked whether the degree of kinship was a good predictor of *H. pylori* sequence similarity, as would be expected if transmission in core families were the predominant mode of spread. A matrix of pairwise kinship coefficients was compared by a Mantel test with a similarity matrix of *H. pylori* sequences. The results were not significant (p = 0.14), consistent with an important role for horizontal transmission. In contrast, a significant correlation was detected when, instead of kinship, a matrix was used that coded whether individuals lived in the same household (p = 0.03). Thus, transmission occurred when persons shared the same household, independent of kinship. We also tested individual transmission pathways for a correlation with *H. pylori* sequence similarity. The median sequence similarities for *H. pylori* from mother-offspring pairs was 0.9520, compared with 0.9665 for both sibling-sibling and father-offspring pairs. These values for three different groups of pairs of first degree relatives were not significantly different (Wilcoxon W = 447.5, *p*-value = 0.3016), suggesting that transmission within families is best described as familial rather than vertical.

### Diversity within an individual host

We next examined genetic diversity within a single individual on the basis of comparisons between the paired isolates from corpus and antrum from 47 members of the rural South African families. These paired isolates were identical in most of the members of families 12 (26/38 [68%]) and 13 (4/9 [44%]). Of the remaining 17 individuals, the isolates from antrum and corpus were genetically related in 14 individuals because they shared between three and six alleles. In contrast, the paired isolates from three individuals were unrelated because they shared 0/7 alleles; all three pairs consisted of isolates from different bacterial populations. Thus, mixed infections were quite commonly detected in the South African families, but most of these were with genetically related bacteria.

### Extensive mosaicism in multilocus haplotypes of *H. pylori* from South African family members

**Figure 4:**
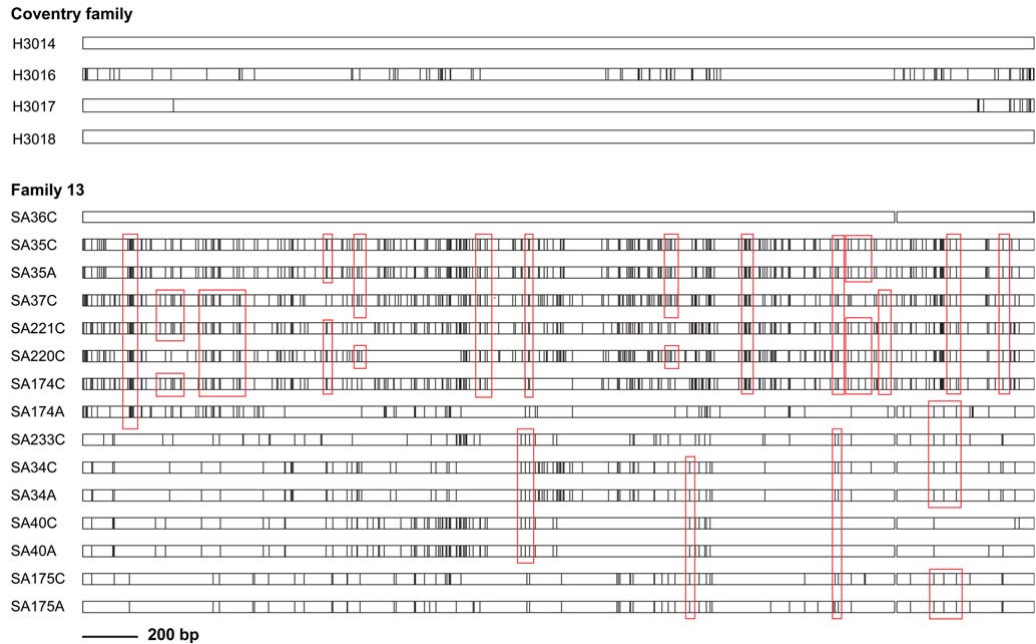
Complex sequence mosaics within *H. pylori* isolates from families. Long bars represent the merged housekeeping gene fragments (3,406 bp) for the strains from the Coventry family (England, top) and family 13 (South Africa, bottom), and black lines within the bars indicate the position of sequence polymorphisms. Gaps indicate a deletion of six basepairs in the *yphC* gene in hpAfrica2 strains. Sequences of paired corpus and antrum isolates from members of family 13 are only shown if the sequences were different. Selected identical sequence motifs, i.e., identical single or multiple nucleotide polymorphisms, detected in isolates from at least two family members of family 13, are highlighted in red.

Recombination during mixed infections with multiple *H. pylori* isolates has previously been identified as the major driving force generating allelic diversity in *H. pylori*. The formation of sequence mosaics is a hallmark of recombination, but nevertheless obvious mosaics have only rarely been described, presumably because recombination is so effective that mosaics rapidly become too fragmented for facile recognition. In order to search for mosaic patterns, multilocus haplotypes were aligned and the positions of sequence differences relative to a guiding sequence were visualized using the program Happlot. Numerous clusters of polymorphic sites that occurred in two or more isolates were readily identified by visual inspection, as shown for two examples in Figure 4. In the Coventry family (England), the grandfather (H3014) and his grandson (H3018) were infected by ST594 isolates. The uncle (H3017) and father (H3016) each carried *H. pylori* with unique STs, but their close genetic relationship to the ST594 isolates is indicated by long stretches of sequence that were identical between all four isolates. Sequence alignments even showed some evidence for recombination in two of the three pairs of antrum and corpus isolates from a South African individual (individuals 172 and 174) where the isolates were from different populations (Figure S1). In contrast, a selection of haplotypes from family 13 shows multiple short clusters of polymorphisms that occur in many different combinations, do not have any clear association with kinship and are responsible for the multitude of individual STs described above.

## Discussion

Almost all earlier studies of *H. pylori* in families were conducted in industrialized countries and analyzed small families consisting of 1^st^ degree relatives only. We therefore anticipated that an analysis of multigeneration families comprising members with varying degrees of kinship might provide additional insights regarding intra-familial transmission of *H. pylori*. We used multilocus sequence haplotyping [2] to analyze the genetic relationships of *H. pylori* isolates from 52 members of two large multi-generation families living in a rural area in South Africa.

### Population analyses

*H. pylori* from the South African families exhibited a striking degree of diversity. Bacteria from four different biogeographical populations (hpAfrica1, hpAfrica2, hpEurope, hpAsia2) circulated among their members. This diversity was unexpected, because these families were from a rural area which should have had little exposure to recent migrant human populations from outside South Africa. All the individuals living within the Ogies communities were born in the area and have continuously resided in this area, with the exception of two individuals (45 and 225 of family 12) who moved to Ogies from other South African towns as adults. However, individuals have migrated to other towns for periods of time in search of work opportunities, and returned later. These individuals or children visiting them may have contributed to the import of non-African *H. pylori* into the community. Alternatively, these different populations have been co-circulating for a long time period, possibly centuries.

The simultaneous circulation of isolates from four populations in a relatively homogeneous African community suggests that there are no major fitness differences between the different populations, especially considering that on rare occasions, bacteria from distinct populations co-infected the same individuals. Otherwise differences in fitness should have led to the rapid disappearance of bacteria that were less fit in such a high prevalence area with frequent mixed infections. We note that hpAfrica2 always lacks the *cag* pathogenicity island [22], which indicates that possession of this pathogenicity island does not increase fitness for colonization.

### Kinship, household, and *H. pylori* transmission

Our analysis of a large global sample indicates that identical multilocus haplotypes are rare in unrelated individuals except in certain rural areas outside of Europe and the Middle East. We interpret the existence of such non-unique STs as evidence for recent horizontal transmission. Pairs or triplets of non-unique STs were found in eight of eleven urban, non-African families (43 individuals), consistent with prior reports showing clonal transmission of *H. pylori* within families [11],[14],[15],[17],[18],[21],[23]. In contrast, all isolates from South African family 13 had unique STs, and only 11 non-unique STs were identified among the 42 members of family 12. Thus, transmission within families was rare in the South African families, although significantly more frequent than in the global sample. Even when less rigorous criteria for genetic relatedness were used, first degree relatives within the non-African urban families were still much more likely to harbor non-unique bacteria than in the South African families or in the global sample. These data suggest that intra-familial transmission between close kin plays a less predominant role in spread of *H. pylori* in rural communities in South Africa and elsewhere than it does in urban locales. If familial transmission were the dominant mode of transfer of *H. pylori*, a strong correlation should exist between the degree of human kinship and the genetic similarity of their infecting *H. pylori*. The extended pedigree of South African family 12 permitted a test of this prediction: the degree of relatedness of the *H. pylori* isolates did not vary with kinship in the South African families, thus providing further evidence against a predominantly familial transmission of *H. pylori* in this rural area.

Despite the absence of a significant correlation between kinship and the similarity of isolates, *H. pylori* from persons living in the same household were more similar than those from individuals that live in different households. This is consistent with the results of a recent study showing that the exposure of household members to an *H. pylori* infected individual with acute gastroenteritis may strongly increase their risk of acquiring *H. pylori* [24].

### Transmission of *H. pylori* in rural vs. urban settings

The data suggest that that familial transmission plays a relatively minor role in the South African community where the two families live. Horizontal transmission pathways, such as through contaminated food, water, or via intensive contact between infants and non-parental caretakers may jointly play a more important role than within-family transmission. Non-parental caretakers are a particularly likely source of non-familial *H. pylori* transmission in the Ogies community, because caretakers will commonly care for children from multiple families. The data from the urban families and from global samples from Europe and the Middle East suggests that improved sanitation and standard of living as well as differences in child care may reduce the risk of horizontal transmission, so that familial transmission becomes dominant.

Our conclusion about the role of horizontal transmission in the South African families is also in agreement with the results of an earlier study performed on the same two South African families with a different approach wherein direct sequencing of three *H. pylori* genes from biopsies was used to model transmission in these families [25]. However, due to the direct sequencing approach, neither haplotypes for individual bacterial isolates nor multiple sequences per individual were available, preventing conclusions about bacterial population diversity, intra-individual heterogeneity, or sequence mosaicism.

### Microbial heterogeneity within individuals

It is a limitation of most studies of *H. pylori* in families, including this one, that bacterial heterogeneity within individuals has not been extensively assessed. By studying two isolates from different gastric regions for the two South African families, we made an effort to explore intra-host diversity. Even this limited approach has provided evidence of widespread mixed infections in families 12 and 13 because the isolates were distinct in 12/38 (32%) and 5/9 (56%), respectively, of the individuals from whom two isolates were available.

Our observations are in agreement with a recent study of a family consisting of two parents born and raised in Algeria and their four children all born in France [17]. For each of these six persons, 9–10 *H. pylori* isolates from two sites of the stomach were characterized by sequence analysis at two loci. Mixed infections were present in all six patients, and these sequences contained mosaic clusters of polymorphic sites in multiple combinations, similar to the results in our study. Interestingly, even with this comprehensive approach, one family member (child 2) had bacteria that were not related to any isolates circulating among the other five family members.

Our study provides evidence that the transmission of *H. pylori* may be far more complex than studies from developed countries have suggested. One possible scenario is that of high turnover of the dominant *H. pylori* strain within a host. However, this high turnover scenario is not supported by studies [26] that investigated sequential isolates of *H. pylori* from patients in Colombia, a high prevalence region [27]. In those studies, the dominant isolate was only rarely replaced by an unrelated *H. pylori*. An alternative scenario may be a better match for the available data: Only one isolate colonizes children chronically, even if they are exposed to multiple *H. pylori* strains. However, exposure to unrelated *H. pylori* strains may result in transient mixed infections, after which the super-infecting isolate is lost due to stronger adaptation of the pre-existing dominant strain to its host. Recombination during the transient mixed infections may lead to a cloud of related recombinants derived from the dominant strain that carry small pieces of intruder DNA, similar to the “quasi-species” interpretation to explain the co-existence of multiple variants of hyper-variable genes in single individuals [28]. This interpretation is also supported by the observation that several shared alleles were found between isolates from families 12 and 13 (Table S3). In further support, the isolates from the families also shared individual alleles with the global sample, usually with isolates from neighboring geographical areas: Cape Town or Namibia for the Ogies families; Venezuela and Colombia for the Colombian families; Houston or Louisiana for the U.S.A. families, and Korea or Japan for the Korean families (Table S4).

Under both scenarios, apparent familial transmission in urban areas is a consequence of lower prevalence and smaller households because secondary infections are rare, and are likely to involve the same or a closely related isolate. In high prevalence/large household rural areas, the pattern would be more similar to horizontal transmission because clonal replacement or the acquisition of genetic diversity through recombination would involve multiple, genetically distinct isolates, resulting in greater diversity between isolates from different individuals.

### Summary

Our study of *H. pylori* transmission, the largest performed to date, demonstrates the potential of using high resolution multilocus sequence haplotype analysis to solve open questions in the epidemiology of this pathogen. Future studies should aim at sampling both a large number of individuals and the intra-host diversity, possibly by metagenomic approaches, but analyses on an even larger scale than that of this study will probably require the development of minimally invasive methods for sampling the stomach flora.

## Materials and Methods

### Bacteria

We analyzed *H. pylori* from 95 individuals in 13 families (Table S5). The isolation of some of these bacteria has been described previously: One isolate was obtained from the antrum or corpus of 37 members of 9 families from Houston, TX, U.S.A.; Seoul, Korea and Bogota, Colombia [21], two members of a family in Belfast, Northern Ireland and four members of a three generation family in Coventry, England [15]. These bacteria had been previously tested by fingerprinting and sequencing of several gene fragments [15],[21]. We also isolated *H. pylori* from biopsies from the corpus and antrum obtained during endoscopy of most members of two multigeneration families in rural South Africa. These families have been followed as part of a long term surveillance program aimed at studying the epidemiology of *H. pylori* within an African community. Ethical approval was obtained from the University of Pretoria and the Hospital Review Board of the Unitas hospital, as previously reported [25], [29]–[31]. We succeeded in obtaining cultivated bacteria from both antrum and corpus from 47 individuals but cultivation was only successful from either antrum or corpus in five other individuals, resulting in 99 isolates from 52 individuals. These two families were from a black community (Ogies, Mpumulanga) living in brick housing with good sanitation and community water supply in a rural area 100 km east of Pretoria, South Africa. The pedigrees of these families are presented in Figures 1 and 2, including 26 members of family 12 and four members of family 13 from whom no isolates were obtained because they were either *H. pylori* negative, deceased, or refused participation in the study.

The global sample consisted partly of published data for 769 isolates from 51 locations. [12]. The data was supplemented with currently unpublished data from multiple analyses in an additional 48 locations where at least two *H. pylori* were isolated, predominantly from native inhabitants of rural areas. Where multiple isolates were available from single individuals, only one was chosen at random. Similarly, only one isolate was chosen at random from known, multiple family members, but this procedure may not have been fully successful because questionnaires were not used systematically to determine family membership. A summary of the geographical sources of all 1,852 such isolates is provided in Table S1.

### PCR and sequencing

Genomic DNA was extracted by using the QIAamp DNA Mini kit (Qiagen). Fragments of seven housekeeping genes (*atpA*, *efp*, *mutY*, *ppa*, *trpC*, *ureI* and *yphC*) were obtained by PCR amplification as previously described [2] and PCR products were purified using the QIAquick PCR Purification kit (Qiagen). Automated sequencing of independent PCR amplicons for each strand was performed with the Big Dye Terminator v1.1 Cycle Sequencing Kit (Applied Biosystems) and either a 3730 XL or 3130*xl* capillary sequencer (Applied Biosystems).

### Phylogenetic analyses and assignment of sequence types

Sequences were assembled, edited and trimmed to a common length using the program BioNumerics 4.0 (Applied Math, Belgium). Allele numbers representing unique sequences were assigned with the Applied Math script “Find and update MLST alleles” and Sequence Type (ST) numbers for each unique allelic profile were assigned with the Applied Math script “Assign ST”. The concatenated sequences from each isolate were assigned to populations using the program STRUCTURE 2.0 [32] as described [3]. Polymorphic blocks of nucleotides were drawn using the program Happlot written by Thomas Whittam (http://www.shigatox.net/cgi-bin/stec/happlot).

### Assignment of kinship coefficients

Pedigree trees, converted into gedcom format with the program GenoPro (V 1.99c; www.genopro.com) were analyzed with the program KStableau (V 4.1.3b; http://home.versatel.nl/KStableau) in order to calculate kinship coefficients for all possible pairs of family members. First degree relatives have a kinship coefficient of 0.25, as based on the 25% chance that both alleles at a single locus from both individuals are from the same source, either paternal or maternal. Similarly, the kinship coefficient for 2nd degree relatives is 0.125, etc. The kinship for unrelated individuals (i.e. individuals from different families) was arbitrarily set to 0.

### Statistical analysis

To quantify the incidence of transmission among first-degree relatives, we estimated the number of isolates with non-unique haplotypes among first-degree relatives (parent-offspring and sibling-sibling) as well as the total number of isolates that had been sampled from these individuals. Similar estimates were performed on the global sample, except that non-unique haplotypes were screened within 97 population samples of at least two individuals. Non-unique haplotypes were scored at various levels of stringency ranging from total identity (7/7 alleles) through partial identity (5 or 6 out of 7 alleles or percentages of homology for concatenated sequences ranging from 99–99.95%).We then compared the frequency of such non-unique isolates in pair-wise comparisons of rural South African families, urban families and the global sample using a χ^2^ test.

The deep pedigree for family 12 in South Africa allowed us to ask more specific questions about transmission among relatives. We correlated pairwise sequence similarity among isolates from all members of the family to the pairwise kinship coefficients using a Mantel test. We then tested whether pairwise similarity of *H. pylori* was greater in members of the same household by creating a matrix in which pairs of individuals from the same household were coded as 0 and pairs from different households were scored as 1. That matrix was then tested against the *H. pylori* sequence similarity matrix. Finally, we compared similarity among three types of close relatives: mother-offspring, father-offspring and sibling-sibling pairs to investigate specific routes of transmission within families.

### Sequence deposition

All unique sequences and their affiliation with individuals have been deposited at http://www.pubmlst.net/Hpylori. A table with allelic profiles and ST numbers for all strains is provided as Table S3.

## Supporting Information

Table S1 Sources of non-familial *H. pylori*
(0.03 MB DOC)

Table S2 Numbers (frequencies) of non-unique non-familial *H. pylori* by continent
(0.04 MB DOC)

Table S3 ST and allele designations for *Helicobacter pylori* isolates from multiple families
(0.06 MB PDF)

Table S4 Global sources of alleles in family as well as in global isolates
(0.04 MB PDF)

Table S5 Sources of *H. pylori* isolates used in this study
(0.14 MB DOC)

Figure S1 Haplotype comparisons between pairs of *H. pylori* strains belonging to different biogeographic populations that colonize antrum and corpus of the same stomach. Long bars represent the merged housekeeping gene fragments (3,406 bp) and black lines within the bars indicate the position of sequence polymorphisms in comparison with a reference strain from the respective family (see Materials and Methods). Gaps indicate a deletion of six basepairs in the *yphC* gene in hpAfrica2 strains. Selected identical sequence motifs, suggestive of recombination between the strains from different populations are highlighted in red, and are detectable in two out of three pairs of strains.
(0.05 MB PDF)
