# Supplementary material for: Horizontal versus Familial Transmission of Helicobacter pylori
Source: PLoS Pathog. 2008 Oct 24;4(10):e1000180. doi: 10.1371/journal.ppat.1000180 (PMC2563686; doi:10.1371/journal.ppat.1000180)
Supplement: Table S1 — Sources of non-familial H. pylori (0.03 MB DOC) [file ppat.1000180.s001.doc]

Table S1. Sources of non-familial *H. pylori*

| Continent | Samples | Number |
| --- | --- | --- |
| Africa | 10 | 241 |
| Americas | 10 | 103 |
| Asia | 36 | 775 |
| Europe | 10 | 176 |
| India | 3 | 62 |
| Middle East | 7 | 107 |
| Pacific Islands | 14 | 157 |
| Sahul | 7 | 231 |
| Total: | 97 | 1852 |
