# Supplementary material for: Horizontal versus Familial Transmission of Helicobacter pylori
Source: PLoS Pathog. 2008 Oct 24;4(10):e1000180. doi: 10.1371/journal.ppat.1000180 (PMC2563686; doi:10.1371/journal.ppat.1000180)
Supplement: Table S2 — Numbers (frequencies) of non-unique non-familial H. pylori by continent (0.04 MB DOC) [file ppat.1000180.s002.doc]

Table S2. Numbers (frequencies) of non-unique non-familial *H. pylori* by continent

| Criterion | Africa | Americas | Asia | Europe | India | Middle East | Pacific Islands | Sahul | Total |
| --- | --- | --- | --- | --- | --- | --- | --- | --- | --- |
| 99.95% | 30 (0.124) | 27 (0.262) | 96 (0.124) | 12 (0.068) | 4 (0.065) | 2 (0.019) | 8 (0.051) | 35 (0.152) | 214 (0.116) |
| 99.9% | 36 (0.149) | 27 (0.262) | 108 (0.139) | 14 (0.080) | 6 (0.097) | 2 (0.019) | 11 (0.070) | 47 (0.203) | 251 (0.136) |
| 99.5% | 51 (0.212) | 29 (0.282) | 154 (0.199) | 16 (0.091) | 9 (0.145) | 4 (0.037) | 23 (0.146) | 64 (0.277) | 350 (0.189) |
| 99.0% | 66 (0.274) | 31 (0.301) | 189 (0.244) | 16 (0.091) | 14 (0.226) | 4 (0.037) | 35 (0.223) | 81 (0.351) | 436 (0.235) |
| 7/7 alleles | 11 (0.046) | 25 (0.243) | 80 (0.103) | 8 (0.045) | 4 (0.065) | 0 (0.000) | 4 (0.025) | 19 (0.082) | 151 (0.082) |
| 6/7 alleles | 37 (0.154) | 29 (0.282) | 114 (0.147) | 14 (0.080) | 6 (0.097) | 2 (0.019) | 16 (0.102) | 44 (0.190) | 262 (0.141) |
| 5/7 alleles | 53 (0.220) | 29 (0.282) | 148 (0.191) | 14 (0.080) | 10 (0.161) | 2 (0.019) | 23 (0.146) | 60 (0.260) | 339 (0.183) |
