# Supplementary material for: Horizontal versus Familial Transmission of Helicobacter pylori
Source: PLoS Pathog. 2008 Oct 24;4(10):e1000180. doi: 10.1371/journal.ppat.1000180 (PMC2563686; doi:10.1371/journal.ppat.1000180)
Supplement: Table S3 — ST and allele designations for Helicobacter pylori isolates from multiple families (0.06 MB PDF) [file ppat.1000180.s003.pdf]

Table S3. ST and allele designations for *Helicobacter pylori* isolates from multiple families

ST	Group	Country	Strain	Allele designation						
				atpA	*efp*	*mutY*	*ppa*	*trpC*	*ureI*	*yphC*
501	Ogies Family 13	South Africa	SA34A	413	415	365	400	414	371	365
502	Ogies Family 12	South Africa	SA162A	320	324	398	414	324	1603	330
502	Ogies Family 12	South Africa	SA162C	320	324	398	414	324	1603	330
503	Ogies Family 13	South Africa	SA175A	841	422	406	422	420	1604	420
504	Ogies Family 12	South Africa	SA302A	1547	510	414	510	421	511	510
504	Ogies Family 12	South Africa	SA302C	1547	510	414	510	421	511	510
505	Ogies Family 13	South Africa	SA40A	1548	540	417	429	1569	288	1552
506	Ogies Family 12	South Africa	SA161A	1549	1464	419	1259	1570	1605	1599
506	Ogies Family 12	South Africa	SA161C	1549	1464	419	1259	1570	1605	1599
507	Ogies Family 12	South Africa	SA160A	1550	1465	425	1495	1571	1606	1600
507	Ogies Family 12	South Africa	SA160C	1550	1465	425	1495	1571	1606	1600
508	Ogies Family 12	South Africa	SA170A	1551	1466	1549	1496	1572	1607	1601
508	Ogies Family 12	South Africa	SA170C	1551	1466	1549	1496	1572	1607	1601
509	Ogies Family 13	South Africa	SA37A	1552	1467	1550	1497	1573	274	1602
509	Ogies Family 13	South Africa	SA37C	1552	1467	1550	1497	1573	274	1602
510	Ogies Family 12	South Africa	SA303A	1553	1468	1551	1498	1574	1608	1603
511	Ogies Family 12	South Africa	SA173A	1554	1469	1552	1499	1575	516	1604
511	Ogies Family 12	South Africa	SA173C	1554	1469	1552	1499	1575	516	1604
512	Ogies Family 13	South Africa	SA233A	1555	1470	1553	429	1576	1609	1605
512	Ogies Family 13	South Africa	SA233C	1555	1470	1553	429	1576	1609	1605
513	Ogies Family 12	South Africa	SA169A	1556	1471	1554	279	1577	315	288
514	Ogies Family 12	South Africa	SA163A1	280	278	294	280	1578	280	295
514	Ogies Family 12	South Africa	SA163C1	280	278	294	280	1578	280	295
514	Ogies Family 12	South Africa	SA210A1	280	278	294	280	1578	280	295
514	Ogies Family 12	South Africa	SA210C1	280	278	294	280	1578	280	295
514	Ogies Family 12	South Africa	SA300A	280	278	294	280	1578	280	295
514	Ogies Family 12	South Africa	SA300C	280	278	294	280	1578	280	295
514	Ogies Family 12	South Africa	SA31C	280	278	294	280	1578	280	295
515	Ogies Family 13	South Africa	SA174A	1557	1472	1555	1500	1579	1610	1606
516	Ogies Family 13	South Africa	SA221A	291	273	1556	291	1580	273	273
516	Ogies Family 13	South Africa	SA221C	291	273	1556	291	1580	273	273
517	Ogies Family 13	South Africa	SA34C	413	415	365	400	414	1611	365

For families from Ogies, the strain designation includes A for antrum or C for corpus. In some cases, additional numbers indicate which of multiple single colonies was chosen

Because only one isolate was available per individual from other families, the strain designation there is the same as the individual designation

ST numbers are cognate with Figures 1-3.

Alleles that were present in isolates from other global sources are indicated in red

Table S3. ST and allele designations for *Helicobacter pylori* isolates from multiple families

ST	Group	Country	Strain	Allele designation						
				atpA	*efp*	*mutY*	*ppa*	*trpC*	*ureI*	*yphC*
518	Ogies Family 12	South Africa	SA303C	1553	1468	1557	1498	1574	1612	1603
519	Ogies Family 12	South Africa	SA169C	1556	1471	1554	279	1577	1613	288
520	Ogies Family 13	South Africa	SA40C	1548	540	417	429	1569	288	1607
521	Ogies Family 13	South Africa	SA220A	320	279	1558	296	1581	279	279
521	Ogies Family 13	South Africa	SA220C	320	279	1558	296	1581	279	279
522	Ogies Family 12	South Africa	SA301C	320	1473	1559	414	324	284	330
523	Ogies Family 13	South Africa	SA174C	291	273	273	1501	1582	273	273
524	Ogies Family 13	South Africa	SA35C	280	278	1560	278	1583	1614	306
525	Ogies Family 13	South Africa	SA175C	1558	422	1561	422	420	1615	420
526	Ogies Family 13	South Africa	SA36C	1559	1474	1562	1502	1584	288	1608
527	Ogies Family 12	South Africa	SA166A	1560	1475	1563	279	1585	288	1609
528	Ogies Family 12	South Africa	SA301A	320	1473	1564	414	324	284	330
529	Ogies Family 13	South Africa	SA35A	280	278	1560	278	1583	1616	306
530	Ogies Family 12	South Africa	SA171A1	515	510	511	510	511	511	510
530	Ogies Family 12	South Africa	SA171C1	515	510	511	510	511	511	510
530	Ogies Family 12	South Africa	SA172A1-1	515	510	511	510	511	511	510
533	Houston, TX	USA	H1	411	413	411	412	412	413	417
534	Ogies Family 12	South Africa	SA226	1561	1476	577	571	1586	1617	304
535	Houston, TX	USA	H2-4	412	414	412	201	269	414	418
536	Houston, TX	USA	H2	205	200	413	413	413	415	419
536	Houston, TX	USA	H2-1	205	200	413	413	413	415	419
537	Houston, TX	USA	H3-1	514	507	508	505	513	516	519
538	Houston, TX	USA	H3	414	416	415	415	415	416	421
539	Houston, TX	USA	H1-1	415	417	416	416	416	417	422
540	Houston, TX	USA	H2-2	416	200	413	413	413	415	419
541	Houston, TX	USA	H2-3	205	200	413	413	413	418	419
542	Houston, TX	USA	H1-2	417	418	663	417	417	419	423
543	Bogota	Colombia	C5	418	419	418	226	418	420	424
544	Bogota	Colombia	C5-1	419	420	728	266	419	421	425
545	Bogota	Colombia	C5-2	420	268	420	418	1546	420	426
546	Bogota	Colombia	C5-3	421	268	420	418	1546	420	426
546	Bogota	Colombia	C5-4	421	268	420	418	1546	420	426

Table S3. ST and allele designations for *Helicobacter pylori* isolates from multiple families

ST	Group	Country	Strain	Allele designation						
				atpA	*efp*	*mutY*	*ppa*	*trpC*	*ureI*	*yphC*
547	Bogota	Colombia	C6	422	421	421	228	705	422	427
548	Bogota	Colombia	C6-1	423	421	422	228	705	422	427
549	Bogota	Colombia	C6-2	424	1448	423	419	422	423	428
550	Seoul	Korea	K3	425	423	424	420	423	424	429
551	Seoul	Korea	K3-1	426	424	44	421	424	425	430
551	Seoul	Korea	K3-2	426	424	44	421	424	425	430
551	Seoul	Korea	K3-4	426	424	44	421	424	425	430
552	Bogota	Colombia	C7-1	427	425	1258	252	425	426	431
553	Seoul	Korea	K1-2	428	426	426	968	426	427	432
554	Seoul	Korea	K1-4	429	427	427	423	427	428	433
555	Seoul	Korea	K5-2	54	428	428	424	428	429	434
555	Seoul	Korea	K5-3	54	428	428	424	428	429	434
556	Bogota	Colombia	C7-3	430	425	1258	252	425	426	431
557	Seoul	Korea	K1	431	429	429	425	429	430	435
557	Seoul	Korea	K1-1	431	429	429	425	429	430	435
558	Bogota	Colombia	C7	423	430	430	228	430	431	436
558	Bogota	Colombia	C7-2	423	430	430	228	430	431	436
559	Seoul	Korea	K5-1	54	428	428	424	428	432	437
560	Seoul	Korea	K5	54	431	428	424	428	433	434
561	Seoul	Korea	K1-3	432	432	431	426	431	434	438
562	Seoul	Korea	K3-3	426	433	44	421	424	425	430
563	Seoul	Korea	K3-5	426	424	44	421	424	435	430
564	Ogies Family 12	South Africa	SA30A1	295	278	1565	299	280	267	1610
564	Ogies Family 12	South Africa	SA30C1	295	278	1565	299	280	267	1610
565	Ogies Family 12	South Africa	SA29A1	1562	1475	1566	429	1569	1608	1611
566	Ogies Family 12	South Africa	SA222A1	1563	1477	1567	1503	1587	1618	1612
566	Ogies Family 12	South Africa	SA222C1	1563	1477	1567	1503	1587	1618	1612
567	Ogies Family 12	South Africa	SA164A1	1547	510	511	510	511	511	510
567	Ogies Family 12	South Africa	SA164C1	1547	510	511	510	511	511	510
567	Ogies Family 12	South Africa	SA165A1	1547	510	511	510	511	511	510
567	Ogies Family 12	South Africa	SA165C1	1547	510	511	510	511	511	510
568	Ogies Family 12	South Africa	SA224A1	1564	1478	513	279	324	527	512

Table S3. ST and allele designations for *Helicobacter pylori* isolates from multiple families

ST	Group	Country	Strain	Allele designation						
				atpA	*efp*	*mutY*	*ppa*	*trpC*	*ureI*	*yphC*
568	Ogies Family 12	South Africa	SA224C1	1564	1478	513	279	324	527	512
568	Ogies Family 12	South Africa	SA29C1	1564	1478	513	279	324	527	512
569	Ogies Family 12	South Africa	SA253A1	1565	1479	1568	1504	1588	288	1613
569	Ogies Family 12	South Africa	SA253C1	1565	1479	1568	1504	1588	288	1613
570	Ogies Family 12	South Africa	SA158A1	280	278	294	280	1589	280	295
571	Ogies Family 12	South Africa	SA46A1	1566	1480	294	267	280	1619	1614
571	Ogies Family 12	South Africa	SA46C1	1566	1480	294	267	280	1619	1614
572	Ogies Family 12	South Africa	SA168A1	1564	1478	513	279	324	1620	512
572	Ogies Family 12	South Africa	SA168C1	1564	1478	513	279	324	1620	512
573	Ogies Family 12	South Africa	SA146A1	1567	1481	297	1505	1590	1621	1615
573	Ogies Family 12	South Africa	SA146C1	1567	1481	297	1505	1590	1621	1615
574	Ogies Family 12	South Africa	SA252A1	320	1482	513	279	324	1622	525
575	Ogies Family 12	South Africa	SA227A1	320	324	324	414	324	1623	330
576	Ogies Family 12	South Africa	SA156A1	1568	1466	1569	303	1591	1624	320
576	Ogies Family 12	South Africa	SA156C1	1568	1466	1569	303	1591	1624	320
577	Ogies Family 12	South Africa	SA45A1	1567	1483	1570	1506	280	1625	1616
578	Ogies Family 12	South Africa	SA144A1	1569	1484	1571	279	1592	1626	1617
579	Ogies Family 12	South Africa	SA214A1	305	1464	1572	1259	341	1605	1599
579	Ogies Family 12	South Africa	SA214C1	305	1464	1572	1259	341	1605	1599
579	Ogies Family 12	South Africa	SA215C1	305	1464	1572	1259	341	1605	1599
579	Ogies Family 12	South Africa	SA216A1	305	1464	1572	1259	341	1605	1599
579	Ogies Family 12	South Africa	SA216C1	305	1464	1572	1259	341	1605	1599
580	Ogies Family 12	South Africa	SA47A1	1570	1485	1573	1507	1593	1627	1618
580	Ogies Family 12	South Africa	SA47C1	1570	1485	1573	1507	1593	1627	1618
581	Ogies Family 12	South Africa	SA155A1	1571	1486	1574	1508	1594	1628	1619
581	Ogies Family 12	South Africa	SA155C1	1571	1486	1574	1508	1594	1628	1619
582	Ogies Family 12	South Africa	SA213A1	271	520	271	1509	1595	334	1620
583	Ogies Family 12	South Africa	SA194A1	1572	1487	278	1510	1596	1629	1606
583	Ogies Family 12	South Africa	SA194C1	1572	1487	278	1510	1596	1629	1606
584	Ogies Family 12	South Africa	SA144C1	1569	1484	1571	279	1592	1630	1617
585	Ogies Family 12	South Africa	SA227C1	320	324	324	414	324	992	330
586	Ogies Family 12	South Africa	SA172C1	1573	1488	1575	279	1597	288	1621

Table S3. ST and allele designations for *Helicobacter pylori* isolates from multiple families

ST	Group	Country	Strain	Allele designation						
				atpA	*efp*	*mutY*	*ppa*	*trpC*	*ureI*	*yphC*
587	Ogies Family 12	South Africa	SA157A1	1574	1489	1576	1511	1598	267	1622
587	Ogies Family 12	South Africa	SA157C1	1574	1489	1576	1511	1598	267	1622
588	Ogies Family 12	South Africa	SA45C1	1567	1483	1570	1506	280	1631	1616
589	Ogies Family 12	South Africa	SA158C1	280	278	277	280	1578	280	295
590	Ogies Family 12	South Africa	SA252C1	529	324	513	279	324	527	512
591	Ogies Family 12	South Africa	SA251C1	1575	1484	1571	279	1599	1632	1617
592	Ogies Family 12	South Africa	SA213C1	271	520	271	1509	1595	1633	1620
593	Ogies Family 12	South Africa	SA251A1	1575	1484	1571	279	1592	1628	1617
594	Coventry	UK	H3014	433	434	432	427	433	438	439
594	Coventry	UK	H3018	433	434	432	427	433	438	439
595	Coventry	UK	H3016	434	435	433	427	434	438	440
596	Coventry	UK	H3017	435	434	432	427	433	438	441
597	Northern Ireland	UK	H3022	436	436	434	428	435	439	442
597	Northern Ireland	UK	H3023	436	436	434	428	435	439	442
