# Supplementary material for: Horizontal versus Familial Transmission of Helicobacter pylori
Source: PLoS Pathog. 2008 Oct 24;4(10):e1000180. doi: 10.1371/journal.ppat.1000180 (PMC2563686; doi:10.1371/journal.ppat.1000180)
Supplement: Table S4 — Global sources of alleles in family as well as in global isolates (0.04 MB PDF) [file ppat.1000180.s004.pdf]

Table S4. Global sources of alleles in family as well as in global isolates

Family Source	Allele (# of family STs)	Global sources: # of STs
Bogota, Colombia		
	efp-1448: (1)	Venezuela: 1
	efp-268: (2)	Capetown, South Africa: 1; Venezuela: 1
	mutY-1258: (2)	Philippines: 1; Venezuela: 2
	mutY-728: (1)	Colombia: 1
	ppa-226: (1)	Colombia: 2
	ppa-228: (3)	Colombia: 1
	ppa-252: (2)	Colombia: 1
	ppa-266: (1)	Philippines: 1; Venezuela: 1
	trpC-1546: (2)	Venezuela: 1
	trpC-705: (2)	Spain: 1
	Total ST sources:	Venezuela: 6; Colombia: 5; Philippines: 2; Spain: 1; Capetown: 1
Coventry or Belfast, UK		
	None	
Houston, TX, U.S.A.		
	atpA-205: (2)	Louisiana: 1
	atpA-514: (1)	Houston: 2
	efp-200: (3)	Louisiana: 3
	efp-507: (1)	Houston: 2
	mutY-508: (1)	Houston: 2
	mutY-663: (1)	Philippines: 1; Spain: 1
	ppa-201: (1)	Louisiana: 1
	ppa-505: (1)	Houston: 2
	trpC-269: (1)	Capetown, South Africa: 1
	trpC-513: (1)	Houston: 1
	ureI-516: (1)	Houston: 3
	yphC-519: (1)	Houston: 2
	Total ST sources:	Houston: 14; Louisiana: 5; Philippines: 1; Spain: 1; Capetown: 1
Soeul, Korea		
	atpA-54: (3)	Korea: 1
	mutY-44: (3)	Japan: 1
	ppa-968: (1)	Germany: 1; Houston: 1
	Total ST sources:	Korea: 1; Japan: 1; Kazakhstan: 1; Germany: 1; Houston: 1
Ogies Family 12		
	atpA-271: (2)	Capetown, South Africa: 2
	atpA-280: (3)	Capetown, South Africa: 1
	atpA-295: (1)	Capetown, South Africa: 5
	atpA-305: (1)	Capetown, South Africa: 6
	atpA-320: (6)	Capetown, South Africa: 2; Namibia: 1; Nigeria: 1
	efp-278: (4)	Capetown, South Africa: 2
	efp-324: (4)	Capetown, South Africa: 1
	mutY-271: (2)	Capetown, South Africa: 2
	mutY-277: (1)	Capetown, South Africa: 2
	mutY-278: (1)	Capetown, South Africa: 1
	mutY-294: (3)	Capetown, South Africa: 3
	mutY-297: (1)	Capetown, South Africa: 1
	mutY-324: (2)	Capetown, South Africa: 3
	mutY-577: (1)	Namibia: 1; Australia: 1

Family Source	Allele (# of family STs)	Global sources: # of STs
	ppa-267: (1)	Capetown, South Africa: 1
	ppa-279: (12)	Capetown, South Africa: 2
	ppa-280: (3)	Capetown, South Africa: 2
	ppa-299: (1)	Capetown, South Africa: 4
	ppa-303: (1)	Capetown, South Africa: 4
	ppa-571: (1)	Australia: 1
	trpC-280: (4)	Capetown, South Africa: 2
	trpC-324: (9)	Capetown, South Africa: 1
	trpC-341: (1)	Capetown, South Africa: 1
	ureI-267: (2)	Capetown, South Africa: 1
	ureI-280: (3)	Capetown, South Africa: 1
	ureI-284: (2)	Capetown, South Africa: 2
	ureI-288: (3)	Capetown, South Africa: 1
	ureI-315: (1)	Capetown, South Africa: 1
	ureI-334: (1)	Capetown, South Africa: 1
	ureI-516: (1)	Houston: 3
	ureI-992: (1)	Namibia: 4
	yphC-288: (2)	Capetown, South Africa: 1
	yphC-295: (3)	Capetown, South Africa: 1
	yphC-304: (1)	Capetown, South Africa: 1
	yphC-320: (1)	Capetown, South Africa: 3
	yphC-330: (5)	Capetown, South Africa: 2
	Total ST sources:	Capetown: 63; Namibia: 11; Houston: 3; Australia: 2; Nigeria: 1
Ogies Family 13		
	atpA-280: (2)	Capetown, South Africa: 1
	atpA-291: (2)	Capetown, South Africa: 1
	atpA-320: (1)	Capetown, South Africa: 2; Namibia: 1; Nigeria: 1
	efp-273: (2)	Capetown, South Africa: 1
	efp-278: (2)	Capetown, South Africa: 2
	efp-279: (1)	Capetown, South Africa: 7
	mutY-273: (1)	Capetown, South Africa: 2
	ppa-278: (2)	Capetown, South Africa: 2
	ppa-291: (1)	Capetown, South Africa: 1
	ppa-296: (1)	Capetown, South Africa: 2
	ureI-273: (2)	Capetown, South Africa: 1
	ureI-274: (1)	Capetown, South Africa: 1
	ureI-279: (1)	Capetown, South Africa: 1
	ureI-288: (3)	Capetown, South Africa: 1
	yphC-273: (2)	Capetown, South Africa: 2
	yphC-279: (1)	Capetown, South Africa: 5
	yphC-306: (2)	Capetown, South Africa: 1
	Total ST sources:	Capetown: 33; Namibia: 1; Nigeria: 1
