# Supplementary material for: Horizontal versus Familial Transmission of Helicobacter pylori
Source: PLoS Pathog. 2008 Oct 24;4(10):e1000180. doi: 10.1371/journal.ppat.1000180 (PMC2563686; doi:10.1371/journal.ppat.1000180)
Supplement: Table S5 — Sources of H. pylori isolates used in this study (0.14 MB DOC) [file ppat.1000180.s005.doc]

Table S5: Sources of *H. pylori* isolates used in this study.

| **Source of strain Patient/Isolate identifyer** | **Age** | **Relationship** |
| --- | --- | --- |
|  |  |  |
| **Houston (USA)** |  |  |
| **Family H1** |  |  |
| H1 | 57 | mother |
| H1-1 | 33 | daughter |
| H1-2 | 30 | son |
|  |  |  |
| **Family H2** |  |  |
| H2 | 73 | mother |
| H2-1 | 60 | sister |
| H2-2 | 70 | brother |
| H2-3 | 78 | brother |
| H2-4 | 52 | son |
|  |  |  |
| **Family H3** |  |  |
| H3 | 70 | mother |
| H3-1 | 37 | son |
|  |  |  |
| **Seoul (Korea)** |  |  |
| **Family K1** |  |  |
| K1 | 67 | father |
| K1-1 | 33 | daughter |
| K1-2 | 43 | son |
| K1-3 | 30 | son |
| K1-4 | 41 | son |
|  |  |  |
| **Family K3** |  |  |
| K3 | 74 | mother |
| K3-1 | 32 | son |
| K3-2 | 36 | daughter |
| K3-3 | 38 | daughter |
| K3-4 | 46 | daughter |
| K3-5 | 48 | son |
|  |  |  |
| **Family K5** |  |  |
| K5 | 56 | mother |
| K5-1 | 31 | son |
| K5-2 | 23 | son |
| K5-3 | 25 | daughter |
|  |  |  |
| **Bogota (Colombia)** |  |  |
| **Family C5** |  |  |
| C5 | 64 | mother |
| C5-1 | 21 | daughter |
| C5-2 | 32 | daughter |
| C5-3 | 30 | son |
| C5-4 | 28 | son |
|  |  |  |

| **Family C6** |  |  |  | |
| --- | --- | --- | --- | --- |
| C6 | 77 | father |  | |
| C6-1 | 45 | daughter |  | |
| C6-2 | 48 | son |  | |
|  |  |  |  | |
| **Family C7** |  |  |  | |
| C7 | 74 | mother |  | |
| C7-1 | 35 | daughter |  | |
| C7-2 | 37 | daughter |  | |
| C7-3 | 33 | daughter |  | |
|  |  |  |  | |
| **Belfast (Northern Ireland)** |  |  |  | |
| **Family Belfast** |  |  |  | |
| H3022 | 10 | son |  | |
| H3023 | 34 | father |  | |
|  |  |  |  | |
| **Coventry (England)** |  |  |  | |
| **Family Coventry** |  |  |  | |
| H3018 | 18 | grandson |  | |
| H3014 | 71 | grandfather |  | |
| H3016 | 46 | father |  | |
| H3017 | 50 | uncle |  | |
|  |  |  |  | |
| **Ogies, Mpumulanga (South Africa)** |  |  |  | |
| **Family 12 (antrum and corpus isolates)** |  |  |  | |
| SA213A,C | 21 | daughter |  | |
| SA214A,C | 14 | son |  | |
| SA215C | 7 | daughter (twin) |  | |
| SA216A,C | 7 | daughter (twin) |  | |
| SA45A,C | 51 | husband of SA46 |  | |
| SA46A,C | 49 | mother |  | |
| SA47A,C | 17 | son |  | |
| SA194A,C | 49 | mother |  | |
| SA166A | 63 | daughter |  | |
| SA168A,C | 39 | granddaughter |  | |
| SA169A,C | 46 | daughter |  | |
| SA170A,C | 11 | granddaughter |  | |
| SA173A,C | 21 | grandson |  | |
| SA171A,C | 6 | great-granddaughter |  | |
| SA172A1,A2,C | 7 | great-granddaughter |  | |
| SA303A,C | Unknown | great-granddaughter |  | |
| SA160A,C | 8 | daughter |  | |
| SA161A,C | 14 | grandson |  | |
| SA162A,C | 11 | grandson |  | |
| SA29A,C | 88 | great-grandfather |  | |
| SA30A,C | 55 | daughter |  | |
| SA158A,C | 21 | grandson |  | |
| SA253A,C | 29 | grandson |  | |
| SA300A,C | Unknown | grandson |  | |
| SA31C | 18 | grandson |  | |
| SA226A | 27 | granddaughter |  | |
| SA227A,C | 33 | husband of SA226 |  | |
| SA251A,C | 35 | grandson |  | |
| SA252A,C | 34 | grandson |  | |
| SA163A,C | 11 | great-granddaughter |  | |
| SA210A,C | 5 | great-grandson |  | |
| SA301A,C | Unknown | granddaughter |  | |
| SA302A,C | Unknown | husband of SA301 |  | |
| SA144A,C | 51 | mother |  | |
| SA222A,C | 60 | husband of SA144 |  | |
| SA146A,C | 8 | son |  | |
| SA155A,C | 48 | mother |  | |
| SA156A,C | 9 | daughter |  | |
| SA157A,C | 17 | daughter |  | |
| SA224A,C | 43 | daughter |  | |
| SA164A,C | 4 | grandson |  | |
| SA165A | 5 | grandson |  | |
|  |  |  |  | |
| **Family 13 (antrum and corpus isolates)** |  |  |  | |
| SA35A,C | 80 | father |  | |
| SA36C | 60 | mother |  | |
| SA233A,C | 42 | husband of SA32 |  | |
| SA34A,C | 30 | son |  | |
| SA37A,C | 19 | daughter |  | |
| SA221A,C | 40 | daughter |  | |
| SA220A,C | 46 | husband of SA221 |  | |
| SA174A,C | 12 | granddaughter |  | |
| SA175A,C | 9 | granddaughter |  | |
| SA40A,C | 3 | grandson |  | |
| A, antrum isolate; C, corpus isolate | |  | |  |
