# Supplementary figures and images for: Horizontal versus Familial Transmission of Helicobacter pylori
Source: PLoS Pathog. 2008 Oct 24;4(10):e1000180. doi: 10.1371/journal.ppat.1000180 (PMC2563686; doi:10.1371/journal.ppat.1000180)

Family 12

**29C (hpAfrica1)**

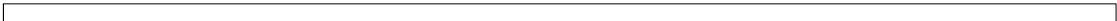

**29A (hpAfrica2)**

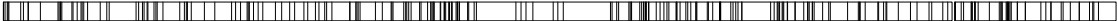

**172A (hpEurope)**

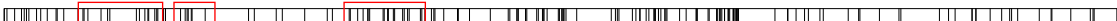

**172C (hpAfrica2)**

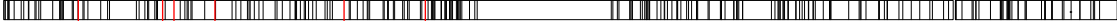

Family 13

**174A (hpAfrica2)**

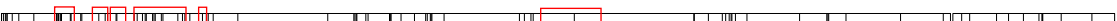

**174C (hpEurope)**

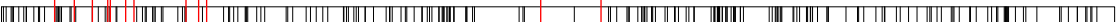

Supplement: Figure S1 — Haplotype comparisons between pairs of H. pylori strains belonging to different biogeographic populations that colonize antrum and corpus of the same stomach. Long bars represent the merged housekeeping gene fragments (3,406 bp) and black lines within the bars indicate the position of sequence polymorphisms in comparison with a reference strain from the respective family (see Materials and Methods). Gaps indicate a deletion of six basepairs in the yphC gene in hpAfrica2 strains. Selected identical sequence motifs, suggestive of recombination between the strains from different populations are highlighted in red, and are detectable in two out of three pairs of strains. (0.05 MB PDF) [file ppat.1000180.s006.pdf]
